# Aspirin‐targeted PD‐L1 in lung cancer growth inhibition

**DOI:** 10.1111/1759-7714.13433

**Published:** 2020-04-15

**Authors:** Yixiang Zhang, Changsheng Lv, Yan Dong, Qingkai Yang

**Affiliations:** ^1^ Department of Thoracic Surgery The First Affiliated Hospital of Dalian Medicine University Dalian China; ^2^ Department of Oncology The First Affiliated Hospital of Dalian Medical University Dalian China; ^3^ Department of Cancer Stem Cell Dalian Medicine University Dalian China

**Keywords:** Aspirin, growth, lung cancer, PD‐L1, TAZ

## Abstract

**Background:**

Aspirin is a classic anti‐inflammatory drug and its anticancer effect has been previously explored in many types of cancer including colorectal cancer therapy. Programmed cell death‐ligand 1 (PD‐L1) is widely expressed in tumor cells and displays an inhibitory role in antitumor immunity. This study aimed to clarify the role of PD‐L1 in aspirin‐suppressed lung cancer.

**Methods:**

The inhibitory effect of aspirin on lung cancer cell proliferation was assessed using an MTT cell viability assay. The role of aspirin in the modulation of PD‐L1 expression was analyzed by western blot or RT‐PCR assays. In lung cancer cells, the influence of aspirin on PD‐L1 promoter activity was detected using a luciferase reporter assay. The interaction of TAZ with PD‐L1 promoter in the cells, with or without aspirin administration, was tested via chromatin immunoprecipitation (ChIP) analysis. The function of PD‐L1 in aspirin‐mediated growth inhibition of lung cancer was examined using a cell viability assay.

**Results:**

Following treatment with aspirin, lung cancer cell growth was markedly suppressed. Aspirin was able to markedly decrease the expression of PD‐L1 at the mRNA and protein levels in lung cancer cells. For the mechanism study, we found that the promoter of PD‐L1 was inactivated by aspirin via TAZ transcriptional coactivator in the cells. With regard to the functional investigation, aspirin was capable of resisting cell proliferation and PD‐L1 overexpression abolished aspirin‐depressed cell proliferation in lung cancer.

**Conclusions:**

Aspirin suppressed the growth of lung cancer cells via targeting the TAZ/PD‐L1 axis.

## Introduction

Serving as a traditional anti‐inflammatory reagent, aspirin has been widely utilized in anticancer investigation. Several reports have revealed that aspirin has been found to regulate certain transcription factors which affect cell apoptosis, proliferation, migration or other processes.^1^, ^2^, ^3^ Aspirin has also been previously reported to be effective in the prevention and treatment of colorectal cancer therapy, and its anticancer effects have since been widely adopted.^4^, ^5^, ^6^ In colorectal cancer, the reduced risk and metastasis is frequently revealed when patients are treated with aspirin.^7^ In many cancers such as ovarian, prostate, or liver cancers, the anticancer effect of aspirin has been well‐studied.^8^, ^9^, ^10^, ^11^ However, the novel targets of the anticancer function of aspirin remain a research hotspot.

Programmed cell death ligand‐1 (PD‐L1) is a vital immune checkpoint molecule which can manipulate cancer cells to escape immune surveillance via its receptor programmed cell death‐1 (PD‐1).^12^, ^13^ PD‐L1 is a transmembrane protein which is expressed in many different cancers including breast, ovarian, bladder, colon, melanoma and lung cancers.^14^, ^15^, ^16^, ^17^, ^18^ Previous studies have investigated PD‐L1 signaling in cancers and have focused on its implications in tumor immune evasion. Recent work shows that PD‐L1 inside tumor cells can regulate ovarian cancer and melanoma cell growth, pathogenesis and autophagy,^19^ promote bladder cancer proliferation, glycolysis,^15^ and be involved in lung cancer chemoresistance.^20^ However, the function of PD‐L1 in aspirin‐resisted lung cancer remains unclear.

In the present investigation, we clarified the role of PD‐L1 as a novel target in aspirin‐suppressed lung cancer and its potential regulatory mechanism. The growth of lung cancer in vitro is suppressed by aspirin. For the mechanism investigation, aspirin resisted the PD‐L1 transcription by targeting the TAZ transcriptional coactivator, resulting in the blocking of lung cancer cell growth. Therefore, our findings indicate that, therapeutically, aspirin can serve as a potential drug for use in lung cancer.

## Methods

### Cell lines

The lung cancer cell lines including A549 and H1299 were obtained from the American Type Culture Collection (ATCC, USA) and grown in DMEM media (Gibco, USA) adding 10% fetal bovine serum (FBS, Gibco), streptomycin (100 μg/mL) and penicillin (100 U/mL) at 37°C with 5% CO_2_.

### Cell viability analysis

Cell viability was assessed using an MTT assay to measure the results of lung cancer cell proliferation. Cells were seeded in 96‐well plates with 3000 cells/well in at least three replicates. Then, 10 hours later, the confluent monolayers were formed, and the medium was changed to medium containing aspirin for another 24, 36 or 72 hours. We then supplemented 10 μL MTT (5 mg/mL) into each well, and after four hours incubation the medium was discarded and MTT in 150 μL DMSO was added into each well. At OD_490nm,_ the absorbance values were measured via an absorbance reader.

### RNA collection and PCR

Total RNA of lung cancer cell samples was extracted using TRIzol reagent. With regard to each sample, cDNA was reverse transcribed from 1 μg RNA. The levels of mRNA were analyzed through reverse transcription (RT)‐PCR using TransScript First‐Strand cDNA Synthesis SuperMix (TransGen Biotech, China). The relative quantification of the mRNA level was performed by the comparative method (2‐△△Ct), and △Ct value of each sample was the average of triplicates.

### Western blot analysis

Western blot analysis was performed in lung cancer cells with different treatments. After aspirin administration, total proteins were obtained from the different cell groups and the proteins in equal measure from each group were examined. The primary antibodies used in this study, anti‐TAZ or anti‐β‐actin, were purchased from Cell Signaling Technology (USA) or Santa Cruz Biotechnology (USA). The secondary antibodies including goat anti‐rabbit (Abcam, USA) or anti‐mouse antibody (Abcam) were incubated with the blots and visualized through an enhanced ECL chemiluminescent substrate kit (Roche, Switzerland).

### Luciferase reporter gene analysis

The lung cancer cell lines including A549 and H1299 (4 × 10^4^ cells/well) were grown on 24‐well plates. The luciferase reporter pGL3‐PD‐L1 plasmid (100 ng/well) and pRL‐TK plasmid (40 ng/well) (Promega, USA) were cotransfected into the cells adding aspirin (ASA). Then, 48 hours later, all group cells were collected to analyze the luciferase activity via dual‐luciferase reporter assay system (Promega, USA). Each experiment was performed a minimum of three times.

### Chromatin immunoprecipitation (ChIP) analysis

For chromatin immunoprecipitation (ChIP) analysis, EpiQuik chromatin immunoprecipitation kit from Epigentek Group Inc. (Brooklyn, NY) was applied. Anti‐TAZ or normal rabbit IgG (a negative control antibody) was utilized to immunoprecipitated the protein‐DNA complexes. DNA extractions from different groups were subsequently used for PCR analysis.

### Statistical analysis

Each experiment was performed on a minimum of three occasions. A Student's *t*‐test was applied to assess the statistical significance comparing ± SD. The statistically significant differences were considered as follows: nonsignificant, ns; *P < 0.05; **P < 0.01; ***P < 0.001.

## Results

### Growth of lung cancer A549 and H1299 cells in vitro suppressed by aspirin

**Figure 1:**
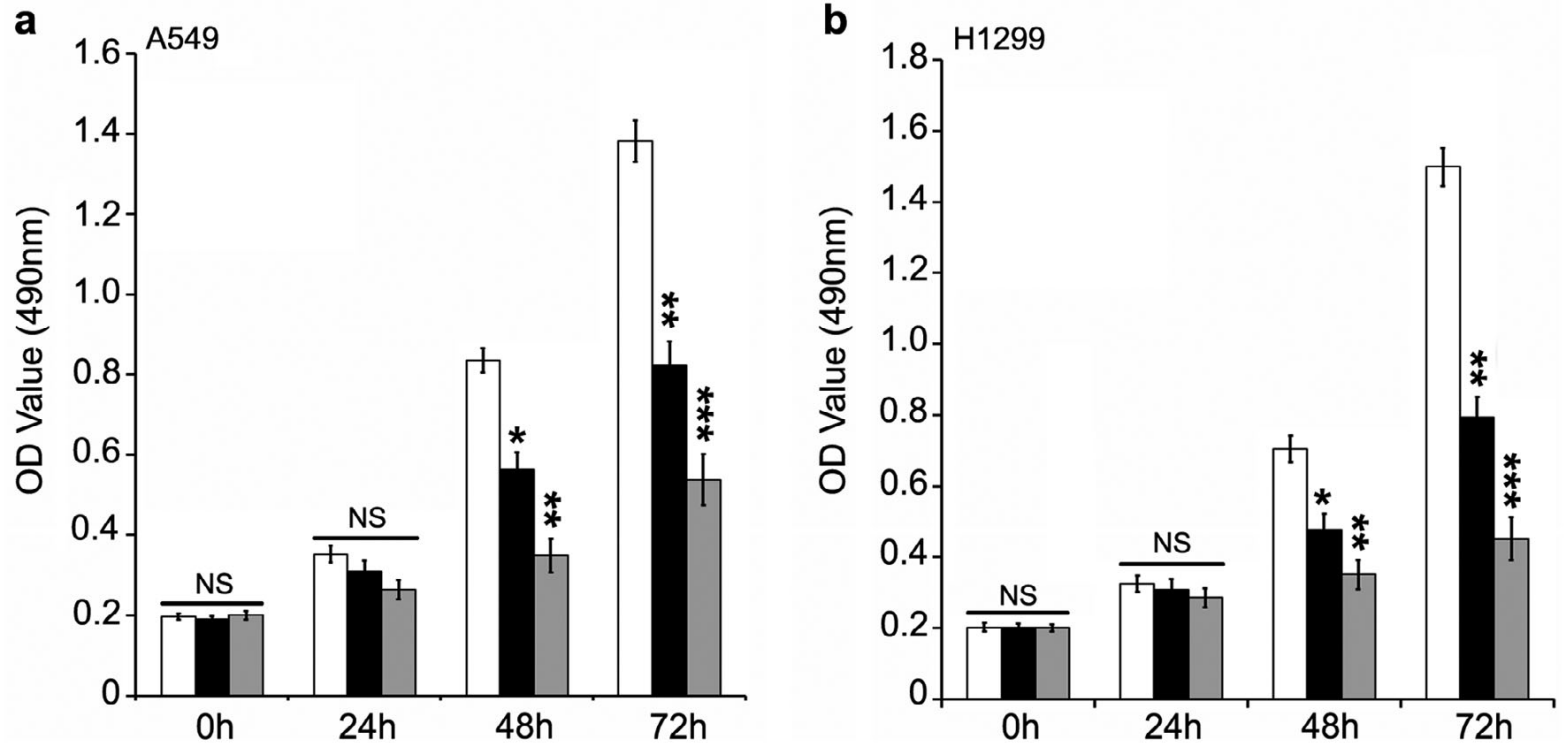
Aspirin (ASA) inhibits the proliferation of lung cancer A549 and H1299 cells in vitro. (**a**,**b**) MTT analysis was used to evaluate the effect of aspirin (ASA) treatment on the proliferation of lung cancer cells including A549 and H1299 (a) (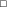) ASA 0 mM, (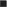) ASA 2.5 mM, (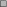) ASA 5.0 mM; (b) (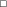) ASA 0 mM, (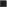) ASA 2.5 mM, (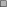) ASA 5.0 mM. ***P < 0.001; **P < 0.01; *P < 0.05. ns, not significant.

Aspirin has been reported to exert an anticancer role in several cancers including colorectal, breast and liver cancers.^6^, ^21^, ^22^, ^23^ However, novel targets in aspirin‐treated lung cancer to a certain extent remain undetermined. To clarify this issue, we first performed cell viability assay in lung cancer cell lines including A549 and H1299, managing the cells with saline or elevated concentrations of aspirin (2.5 mM and 5.0 mM). Our data revealed that the cell viability was obviously suppressed by the elevated concentration of aspirin (Fig 1a,b). We were therefore able to confirm effective suppression of aspirin on the growth of lung cancer cells.

### Decrease of oncogenic PD‐L1 in aspirin‐treated lung cancer cells

**Figure 2:**
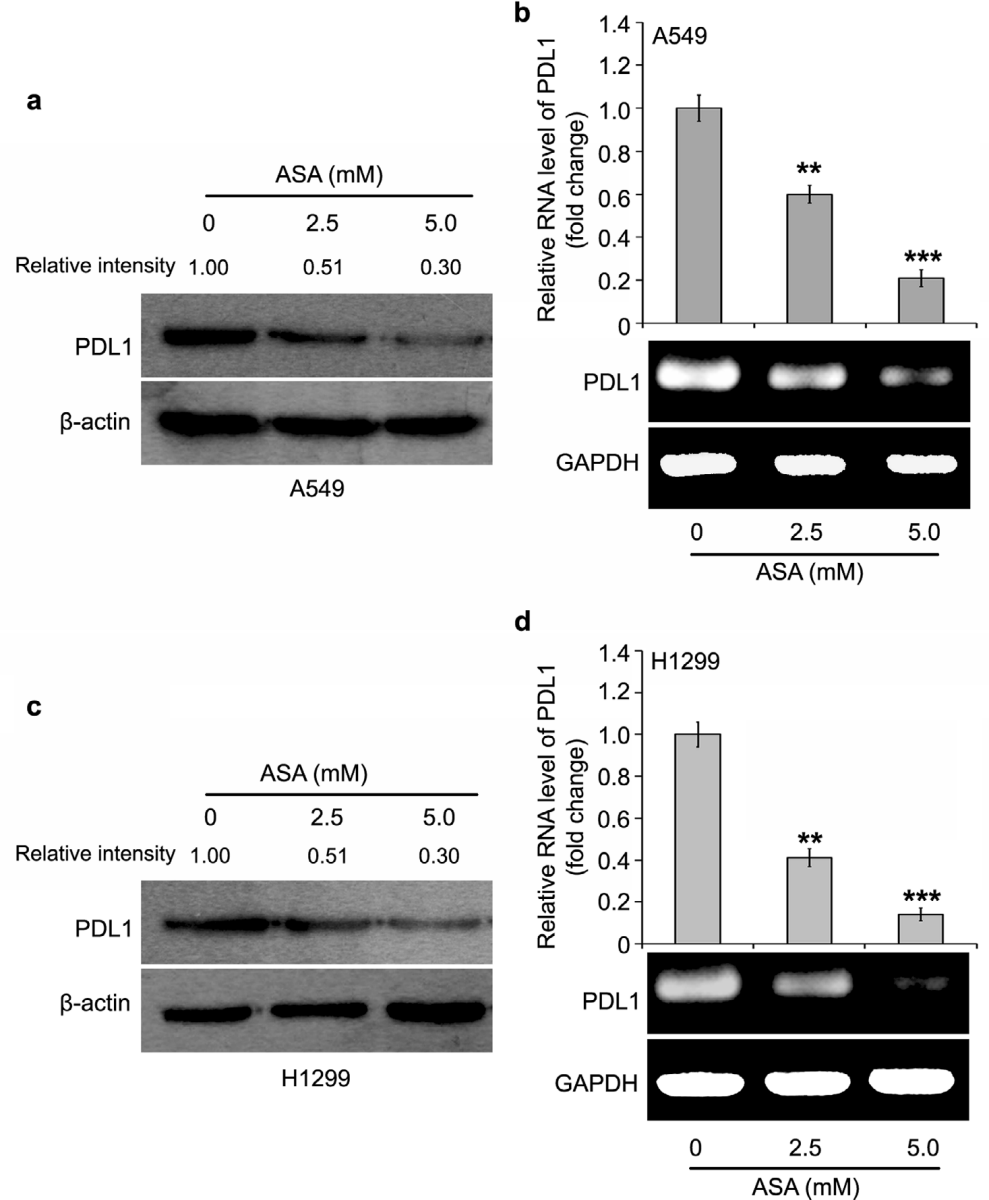
Oncogenic PD‐L1 is decreased in aspirin‐treated lung cancer cells. (**a,b**) Western blot and RT‐PCR assays were performed to test the expression of PD‐L1 at the mRNA and protein levels in the A549 cell line supplemented with aspirin. The quantification of immunoblotting bands was evaluated by BandScan. The statistical change from three independent experiments for RT‐PCR assay is presented. (**c,d**) Western blot and RT‐PCR assays were performed to test the expression of PD‐L1 at the mRNA and protein levels in H1299 cell line supplemented with aspirin. The quantification of immunoblotting bands was evaluated by BandScan. The statistical change from three independent experiments for RT‐PCR assay is presented. ***P < 0.001; **P < 0.01.

PD‐L1 is widely expressed in tumor cells and plays a crucial inhibitory role in antitumor immunity via interaction with receptor PD1 in T lymphocytes. However, whether oncogenic PD‐L1 inside the cells is involved in aspirin‐depressed lung cancer cells is unclear. Using western blot and RT‐PCR analyses, the expression of PD‐L1 at the mRNA and protein level was tested in lung cancer cell lines including A549 and H1299 with the administration of different doses of aspirin. We observed that in contrast to the control group, aspirin was able to significantly reduce the PD‐L1 expression at the protein level in A549 cells (Fig 2a). We further investigated whether the PD‐L1 transcription was affected by aspirin, and our data indicated that the transcription of PD‐L1 was apparently suppressed by treatment with aspirin in the cells (Fig 2b). The data from another lung cancer cell line H1299 was the same as the data from A549 cells (Figs 2c,d). Collectively, our results imply that aspirin was able to control the expression of PD‐L1 in lung cancer cells.

### Aspirin controls PD‐L1 transcription via TAZ transcriptional coactivator

**Figure 3:**
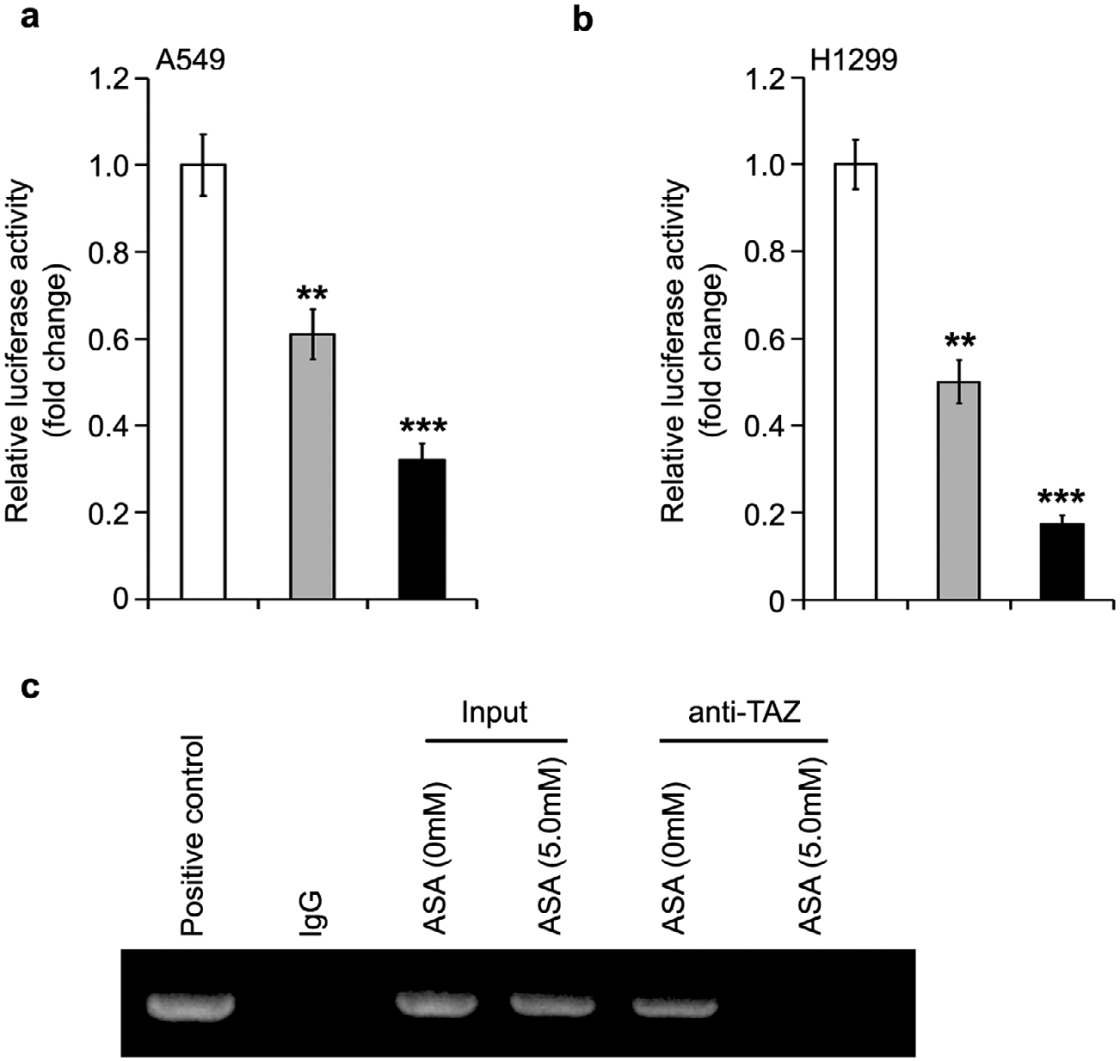
Aspirin controls PD‐L1 transcription via TAZ transcriptional coactivator. (**a,b**) Luciferase reporter gene analysis was used to examine the role of aspirin (ASA) in the regulation of PD‐L1 transcription in A549 and H1299 cell lines (a) (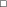) ASA 0 mM, (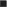) ASA 2.5 mM, (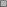) ASA 5.0 mM; (b) (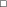) ASA 0 mM, (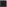) ASA 2.5 mM, (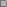) ASA 5.0 mM. ***P < 0.001; **P < 0.01. (**c**) ChIP and PCR assays was used to analyze the interaction of TAZ with PD‐L1 promoter in lung cancer cells with aspirin treatment.

To clarify the molecular mechanism by which aspirin modulates the PD‐L1 transcription in lung cancer, we cloned the core promoter of PD‐L1^24^ into luciferase reporter pGL3‐basic plasmid. Our aim was to determine whether aspirin affected the promoter activities of PD‐L1 in lung cancer cells. We found that the luciferase activity of pGL3‐PD‐L1 was obviously reduced by the treatment with aspirin in A549 cells (Fig 3a). We further confirmed the results using H1299 lung cancer cells (Fig 3b). According to a previous study,^24^ TAZ transcriptional coactivator is able to bind to the promoter of PD‐L1. ChIP assay was used to analyze whether TAZ was involved in aspirin‐controlled PD‐L1 transcription. We found that in lung cancer cells the interaction of TAZ with PD‐L1 promoter was disrupted by aspirin (Fig 3c). Thus, we were able to conclude that aspirin restrains the PD‐L1 transcription by regulating TAZ in lung cancer.

### PD‐L1 serves as a novel target in aspirin‐relieved lung cancer

**Figure 4:**
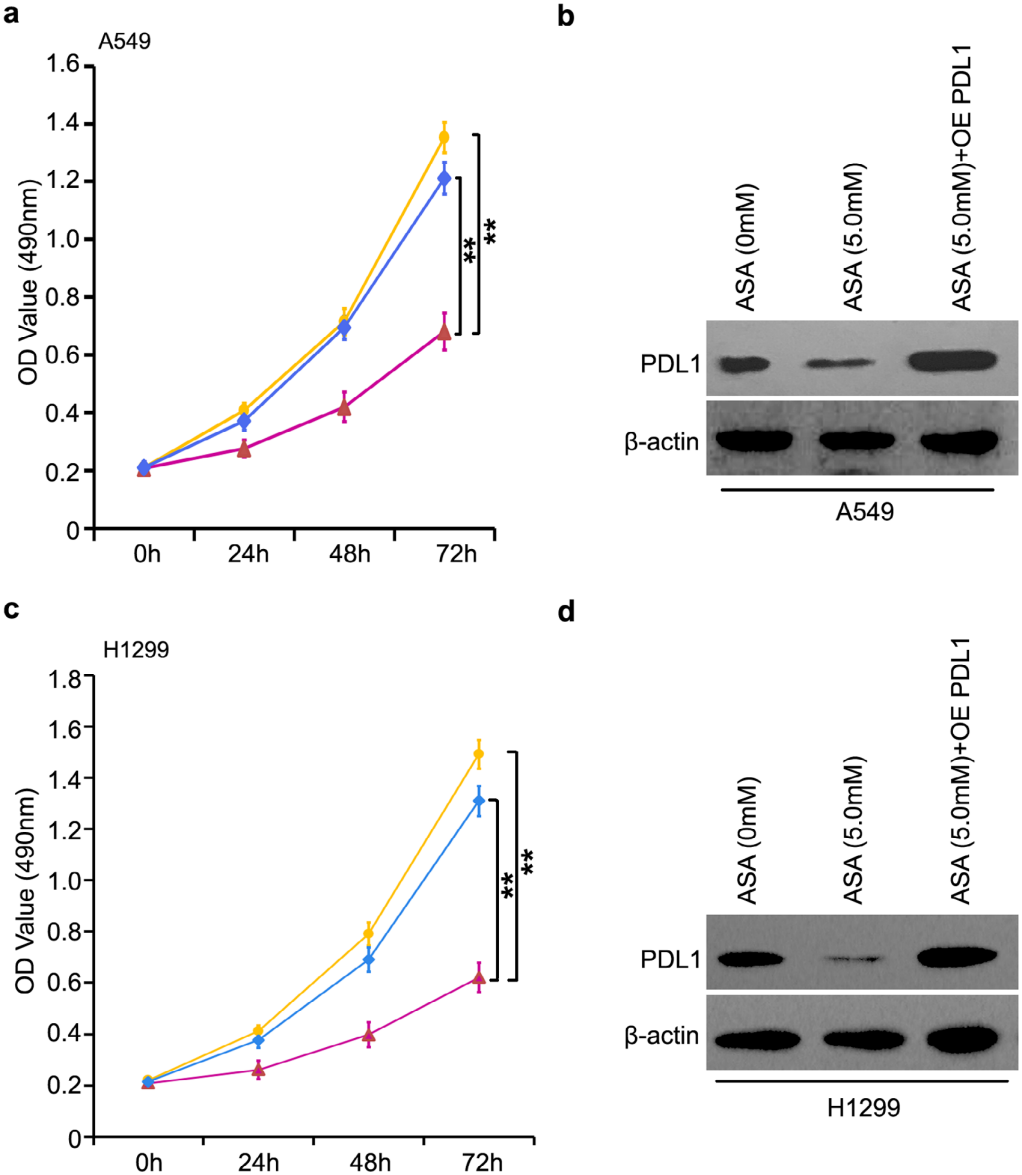
PD‐L1 serves as a novel target in aspirin‐relieved lung cancer. (**a**) The role of aspirin and/or PD‐L1 overexpression (OE PD‐L1) in proliferation was analyzed by MTT assay in the A549 cell line (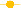) ASA (0 mM), (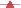) ASA (5.0 mM), (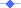) ASA (5.0 mM) + OE PDL1. (**b**) The level of A549 cell line with the treatment of aspirin and/or PD‐L1 overexpression (OE PD‐L1) was examined using western blotting. (**c**) The role of aspirin and/or PD‐L1 overexpression (OE PD‐L1) in proliferation was analyzed by MTT assay in H1299 cell line (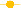) ASA (0 mM), (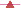) ASA (5.0 mM), (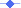) ASA (5.0 mM) + OE PDL1. (**d**) The level of H1299 cell line with the treatment of aspirin and/or PD‐L1 overexpression (OE PD‐L1) was examined using western blotting. **P < 0.01.

Finally, we evaluated the effect of aspirin‐restrained PD‐L1 in the growth of lung cancer cells in vitro. In A549 cells, the addition of 5.0 mM aspirin was able to clearly suppress cell proliferation. Notably, overexpressed PD‐L1 sharply abolished the inhibition of A549 cell proliferation (Fig 4a). Meanwhile, the level of PD‐L1 was evaluated through western blot analysis when A549 cells were treated with aspirin and/or PD‐L1 transfection (Fig 4b). Furthermore, we confirmed that aspirin controlled cell growth and PD‐L1 introduction reversed aspirin‐mediated growth suppression (Fig 4c,d). It was therefore concluded that aspirin suppressed the growth of lung cancer cells via targeting PD‐L1.

## Discussion

Accumulating evidence reveals that aspirin is widely used in colorectal cancer therapy.^4^, ^5^, ^6^ In other cancers such as liver, prostate, or ovarian cancers, the inhibitory effects of aspirin on cancer have also been reported.^8^, ^9^, ^10^, ^11^ However, the novel targets of aspirin in lung cancer therapy remain unexplored.

First, in our study we aimed to confirm the effect of aspirin on the growth of lung cancer. Aspirin was capable of clearly decreasing the cell viability using lung cancer cell lines including A549 and H1299. Serving as a vital immune checkpoint molecule, PD‐L1 can make cancer cells escape immune surveillance.^12^ Most investigations of PD‐L1 signals in cancers have focused on the extrinsic PD‐L1 roles in tumor cells, especially in immune evasion. Recent work shows that tumor‐intrinsic PD‐L1 can regulate ovarian and melanoma cancer growth, pathogenesis and autophagy,^19^ promote bladder cancer proliferation, glycolysis,^15^ and is involved in lung cancer chemoresistance.^20^ Yet, the function of PD‐L1 in aspirin‐resisted lung cancer remains unclear.

In this study, we were interested in the function of PD‐L1 in the growth of aspirin‐suppressed lung cancer. The sharp decrease in PD‐L1 expression at the promoter, mRNA and protein level by aspirin was demonstrated in lung cancer cells. In the next investigation, we will seek to determine how aspirin modulates PD‐L1 in lung cancer cells. A previous study has revealed the role of transcription coactivator TAZ in PD‐L1 transcription,^24^ indicating that TAZ could be involved in aspirin‐controlled PD‐L1 in lung cancer. ChIP assay further confirmed that the interaction of TAZ with PD‐L1 promoter could be disrupted by aspirin. Our final function investigation revealed that PD‐L1 is a novel target in aspirin‐suppressed lung cancer growth in vitro.

In conclusion, our findings identify a new mechanism of relieving lung cancer using aspirin. Aspirin has been shown to be capable of resisting the growth of lung cancer cells. PD‐L1 has been determined to be reduced in aspirin‐inhibited lung cancer. Aspirin regulates the TAZ transcriptional coactivator to suppress PD‐L1 activation, resulting in lung cancer growth blockade. The findings of our study emphasize that aspirin may become a new treatment with great potential in lung cancer therapy.

## Disclosure

No conflict of interest is reported.
